# Pain response of resistance training of the paravertebral musculature under radiotherapy in patients with spinal bone metastases – a randomized trial

**DOI:** 10.1186/1471-2407-14-485

**Published:** 2014-07-05

**Authors:** Harald Rief, Thomas Welzel, Georg Omlor, Michael Akbar, Thomas Bruckner, Stefan Rieken, Matthias F Haefner, Ingmar Schlampp, Alexandros Gioules, Jürgen Debus

**Affiliations:** 1 Department of Radiation Oncology, University Hospital of Heidelberg, Im Neuenheimer Feld 400, Heidelberg 69120, Germany; 2 Department of Medical Biometry, University Hospital of Heidelberg, Im Neuenheimer Feld 305, Heidelberg 69120, Germany; 3 Department of Orthopaedics and Trauma Surgery, University Hospital of Heidelberg, Schlierbacherstrasse 120a, Heidelberg 69118, Germany

**Keywords:** Bone metastases, Spine, Physical exercise, Pain response, Isometric muscle training, Palliative radiotherapy

## Abstract

**Background:**

To compare pain response outcomes for patients with spinal bone metastases treated with resistance training of the spinal musculature versus passive physical therapy during radiotherapy (RT).

**Methods:**

In this randomized trial, 60 consecutive patients were treated from September 2011 until March 2013 within one of the two groups: resistance training (Arm A) or passive physical therapy (Arm B) with thirty patients in each group during RT. The course of pain according to visual analog scale (VAS), concurrent medication, and oral morphine equivalent dose (OMED) were assessed at baseline, three months, and six months after RT. Pain response was determined using International Bone Consensus response definitions.

**Results:**

The course of VAS in the intervention group (Arm A) was significantly lower both during and after RT (AUC, p < .001). The use of analgetic medication showed the same result, with significantly fewer analgetics being necessary both during and after RT in arm A (p < .001). In the course of time, the OMED decreased in arm A, but increased in arm B. After 6 month, 72.2% of patients in arm A, and 22.2% in arm B were responders (p = .014).

**Conclusion:**

Our trial demonstrated that guided isometric resistance training of the paravertebral muscles can improve pain relief over a 6-months period in patients with stable spinal metastases. Importantly, the intervention was able to reduce OMED as well as concomitant pain medication. The trial is registered in Clinical trial identifier NCT 01409720 (http://www.clinicaltrials.gov/) since 2^nd^ of August 2011.

## Background

The spine is the most common site of bone metastases [1,2]. Bone metastases are a major clinical concern and cause severe pain, pathological fractures, spinal cord compression, hypercalcaemia with a significant decrease in quality of life [3]. Pain remains the most frequent symptom and the most important factor impairing the mobility and quality of life in patients with bone metastases [4]. Radiotherapy (RT) is a well-established non-pharmacological effective treatment for the alleviation of pain from spinal metastases [5,6]. Other therapeutic options used to treat pain in addition to RT involve analgetics, systemic therapy, bisphosphonates, and minimal invasive surgery [3]. For these reasons, pain relief is an important clinical challenge and represents the primary goal of any therapy aiming to manage bone metastases [7]. Due to the potentially raised risk of pathological fractures, physicians have so far refrained from initiating forms of intervention involving sport exercises in patients with bone metastases, and there are no specifically exercise-related therapeutic measures involving isometric muscle training described in the literature in connection with bone metastases. Relating to tumor patients of any primary, there are numerous indications of the positive effect of targeted resistance training measures regarding pain and mobility [4,8,9]. Accordingly the effect of muscle-training exercises as an adjunct to RT in patients with bone metastases is still unknown.

The underlying concept of this randomized study is to compare the pain response of an isometric resistance training regimen of the paravertebral muscles as an adjunct to RT vs. RT alone in patients with spinal bone metastases. The objective may be seen as the integration of a combination therapy in palliative-care patients to reduce the primary symptom in cases of spinal bone metastases.

## Methods

### Subjects and recruitment

**Figure 1:**
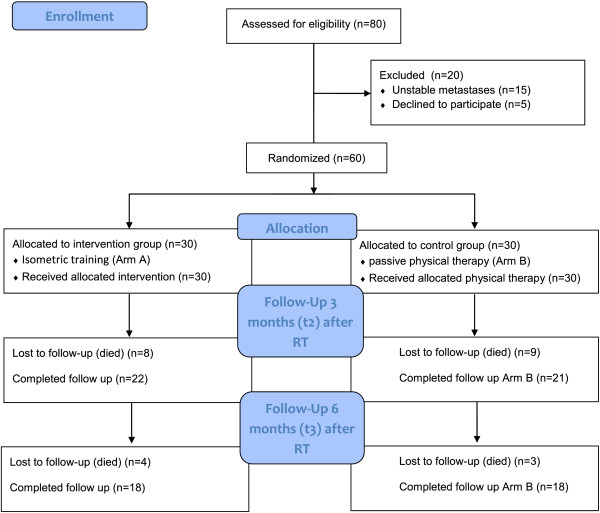
Flow of participants through the trial.

From September 2011 until March 2013, 80 consecutive patients with a histologically confirmed cancer of any primary and solitary or multiple bone metastases of the thoracic or lumbar segments of the vertebral column or of the os sacrum were screened in the Radiooncology Department of the Heidelberg University Clinic. Initially all patients were diagnosed as having painful bone metastases requiring RT. Inclusion criteria were an age of 18 to 80 years, a Karnofsky performance score [10] ≥ 70, written consent to participate, and already initiated bisphosphonate therapy. The patients were subjected to a staging of their vertebral column within the context of the CT designed to plan the radiation schedule prior to enrolment into the trial. In this examination metastases in the thoracic and lumbar spine were classified as “stable” or “unstable”. Patients with stable vertebral-body lesions were included. Out of 80 patients considered eligible, 15 patients were excluded due to unstable metastases, and five patients declined to participate in the study. 60 patients fulfilled the inclusion and were enrolled into the trial (Figure 1). The study was approved by the Heidelberg Ethics Committee (Nr. S-316/2011).

### Design, randomized allocation, and procedures

This is a randomized, controlled, explorative intervention trial to compare pain response of a resistance training program for strengthening the paravertebral muscles in patients with bone metastases as an adjunct to RT. Patients in the control group conducted passive physical therapy in the form of respiratory exercises. A block randomization approach with block size 6 was used to ensure that the two groups were balanced. After baseline measurements, the patients with stable bone metastases were assigned to the respective treatment arms on a 1:1 basis according to the randomization list. The randomization procedure was carried out by a central office. Arm A (intervention group, resistance training) and in Arm B (control group, passive physical therapy) each consisted of 30 patients. The target parameters were measured at the start of radiotherapy (t_0_), end of RT (t_1_), after twelve weeks (t_2_), and after six month (t_3_). The target parameters comprise the documentation of visual analog scale (VAS), neuropathic pain, oral morphine equivalent dose (OMED), concurrent medication, and the recording of patient-specific data. VAS and concurrent medication were documented daily for twelve weeks, and once after 6 month. VAS values following RT were recorded as the mean value for the week. In addition, the basic pain medication and concomitant medication from the start of RT until twelve weeks after RT were recorded daily and also six months after RT. During the therapy the values were documented by the study staff; subsequently the patients themselves continued the documentation in the form of a pain diary. Neuropathic pain was also recorded. The opioid analgetics were converted into an oral morphine equivalent dose (OMED). Non-opioid analgetics were also recorded. The data of the patient records were collected by the authors. Patient characteristics are shown in Table 1.

**Table 1:** Patient characteristics at baseline

		**Intervention group (n = 30)**		**Control group (n = 30)**	
		**n**	**%**	**n**	**%**
Age (years)	Mean (SD)	61.3 (10.1)		64.1 (10.9)	
Gender	Male	14	46.7	19	63.3
	Female	16	53.3	11	36.7
Karnofsky-index (median, range)		80 (70–100)		80 (70–100)	
Primary site					
	Lung cancer	12	40.0	8	26.6
	Breast cancer	5	16.7	6	20.1
	Prostate cancer	5	16.7	9	30.1
	Melanoma	1	3.3	1	3.3
	Renal cancer	1	3.3	2	6.7
	Other	6	20.1	4	13.4
Localization metastases					
	Thoracic	17	56.7	14	46.7
	Lumbar	9	30.0	13	43.3
	Thoracic and lumbar	2	6.7	2	6.7
	Sacrum	2	6.7	1	3.3
Number metastases					
	Mean (range)	1.4 (2-4)		1.7 (1-5)	
	Solitary	22	73.3	18	60.0
	Multiple	8	26.7	12	40.0
Type of metastases					
	Mixed	2	6.7	2	6.7
	Osteoblastic	9	30.0	10	33.3
	Osteolytic	19	63.3	18	60.0
Distant metastases at baseline					
	Visceral	12	40.0	5	16.7
	Brain	3	10.0	3	10.0
	Lung	7	23.3	4	13.3
	Tissue	8	26.7	6	20.0
Hormonotherapy		10	33.3	16	53.3
Immunotherapy		7	23.3	5	16.7
Chemotherapy		25	83.3	20	66.7
Ataractics		4	13.3	2	6.7
NSAR		19	63.3	23	76.7
Opioids		11	36.7	13	43.3

*Abbreviation: SD* standard deviation.

### Study interventions

The interventions commenced on the same day as RT and were performed on each day of RT treatment (Monday through Friday) over a two-week period, independent of the number of fractions. During the two-week RT period, the patients in the resistance training group (Arm A) performed the exercises under guidance of a physiotherapist. The patients were then instructed to practice the training in their homes three times a week and continued the resistance training themselves until the last investigation after six months. The resistance training lasted approx. 30 min, the physical therapy (Arm B) approx. 15 min. Since the site of the bone metastases differed from patient to patient, three different exercises were enacted to ensure an even isometric resistance training of the muscles along the entire vertebral column. The patients of the control group received physical therapy in the form of respiratory exercises also for a period of two weeks. A detailed report of the intervention and its application has already been published [11].

### Measures of the endpoint

The endpoint was pain response, defined according to the International Bone Consensus response categories by Chow et al. [12] as complete response (CR), partial response (PR), pain progression (PP), and stable pain (SP) at three and six months after RT. The pain was documented on the visual analog scale (range 0–100). Complete response was defined as VAS = 0 at treated site with no concomitant increase in analgetic intake (stable or reducing analgetics in daily oral morphine equivalent dose). Partial response was defined as pain reduction of 2 or more at the treated site without analgetic increase, or analgetic reduction of 25% or more from baseline without an increase in pain. Pain progression was defined as increase in pain score of 2 or more above baseline at the treated site with stable OMED, or an increase of 25% or more in OMED compared with baseline with the pain score stable or 1 point above baseline. Any response not covered by the complete response, partial response, or pain progression definitions was called “stable pain” [13]. Responders were defined as CR + PR, non-responders as PP + SP.

### Radiotherapy

RT was performed in the Radiooncology Department of the Heidelberg University Clinic. After virtual simulation was performed to plan the radiation schedule, RT was carried out over a dorsal photon field of the 6MV energy range. PTV covered the specific vertebral body affected as well as the ones immediately above and below. In Arm A 24 patients (80%) were treated with 10 × 3 Gy, three patients (10%) with 14 × 2.5 Gy, and three patients (10%) with 20 × 2 Gy. In Arm B the dose fractions for 28 patients (93.3%) were 10 × 3 Gy, for one patient (3.3%) 14 × 2.5 Gy, and for one patient (3.3%) 20 × 2 Gy. The median individual dose in all patients was 3 Gy (range 2–3 Gy), the median total dose 30 Gy (range 20–35 Gy). The individual and total doses were decided separately for each individual patient, depending on the histology, the patient’s general state of health, and on the current staging and the corresponding prognosis.

### Sample calculation and statistical analysis

The total number of patients undergoing RT in the radiation oncology department of the Heidelberg University Clinic for metastatic processes in the vertebral column in the recruitment period is approx. 120, about 90 of whom shall fulfill the inclusion criteria. On account of the explorative character of this study, it was not possible to estimate the total number of cases; with a scheduled number of 30 patients per group, it will, however, be possible to detect a standardized mean-value effect of 0.8 with a power of 80% and an significance level of 5%. All variables were analyzed descriptively by tabulation of the measures of the empirical distributions. According to the scale level of the variables, means and standard deviations or absolute and relative frequencies, respectively, were reported. Additionally, for variables with longitudinal measurements, the time courses of individual patients and summarized by treatment groups. Descriptive p-values of the corresponding statistical tests comparing the treatment groups will be given. The VAS was adjusted for concurrent medication. Analysis of covariance (ANOVA) with repeated measurements, with group as factor, time (days during RT, weeks after RT), and pain medications as covariance were done. Area under the curve was divided by number of visits per patient. Wilcoxon test was used to detect possible differences between groups. Graphical visualization includes boxplots and means course over time.

## Results

The mean follow-up was 6.3 months for both groups. During the trial there were no adverse events. All surviving patients completed all surveys. Eight patients (26.7%) in Arm A died within the first twelve weeks following RT, additional 4 patients (13.3%) died within 6 months due to tumor progression. In Arm B, 9 patients died (30.0%) within 3 months, and 3 further patients (10.0%) within 6 months. Mortality did not differ between groups.

In arm A, NSAR were taken by 63.3% (n = 19) and opioid analgetics by 36.7% (n = 11) of the patients. In arm B, 76.7% (n = 23) of the patients took NSAR and 43.3% (n = 13) opioids. During the resistance training in arm A 30% (n = 9) of the patients reported resting pain and 46.7% (n = 14) pain upon movement; four of these patients (13.3%) were forced to take relief medication.

**Figure 2:**
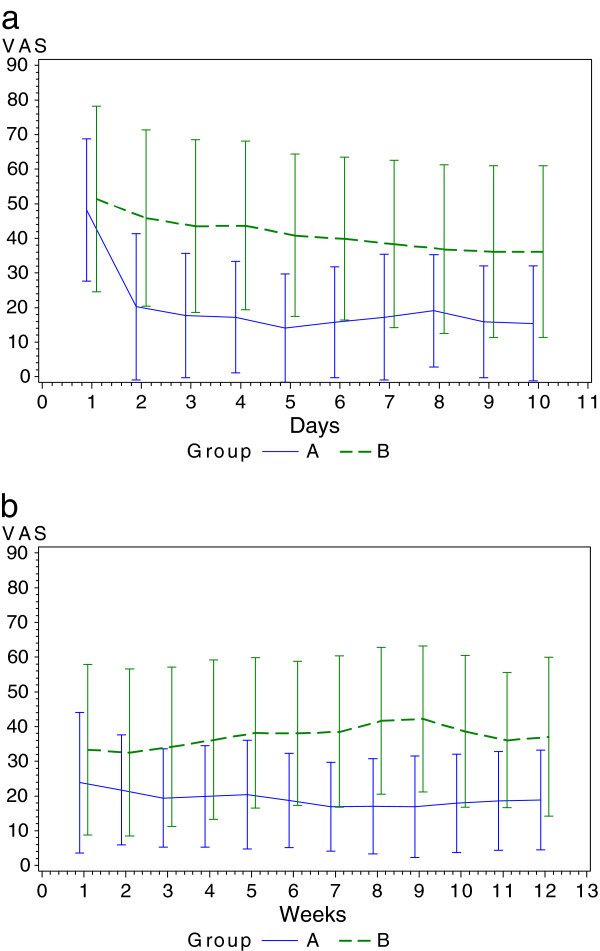
**Visual analog scale during and after RT. a**. VAS course during intervention and RT (T0-T1). **b**. VAS course after intervention and RT (T1-T2).

The course of VAS in the intervention group was significantly lower both during and after RT (AUC, p < .001) (Figure 2a, 2b). The taking of relief medication showed the same result, with significantly fewer analgetics being necessary both during and after RT in arm A (p < .001) (Table 2).

**Table 2:** Results of OMED, VAS, and neuropathic pain

	**Intervention group (n = 30)**			**Control group (n = 30)**			
**OMED**	**n**	**Mean**	**SD**	**n**	**Mean**	**SD**	**p-value**
Baseline (t0)	30	56.8	132.2	30	45.0	86.2	0.841
RT completed (t1)	30	43.8	88.7	30	50.7	88.7	0.452
After 3 months (t2)	22	30.2	59.1	21	62.7	84.6	0.091
After 6 months (t3)	18	20.8	46.9	18	76.7	103.6	0.018
**Visual analog scale**							
Baseline (t0)	30	48.2	20.5	30	51.3	26.9	0.393
RT completed (t1)	30	23.8	20.2	30	33.3	24.6	0.118
After 3 months (t2)	22	15.8	12.1	21	40.7	21.7	<0.001
After 6 months (t3)	18	16.7	14.8	18	50.3	22.8	<0.001
**Neuropathic pain**							
Baseline (t0)	30	0.2	0.4	30	0.2	0.4	0.749
RT completed (t1)	30	0.1	0.3	30	0.2	0.4	0.326
After 3 months (t2)	22	0.2	0.4	21	0.2	0.4	0.619
After 6 months (t3)	18	0.2	0.4	18	0.2	0.4	0.694

VAS was measured from 0 to 100 with 0 indicating no pain at all and 100 indicating worst possible pain.

*Abbreviations: SD* standard deviation, *OMED* oral morphine equivalent dose, *VAS* visual analog scale.

**Figure 3:**
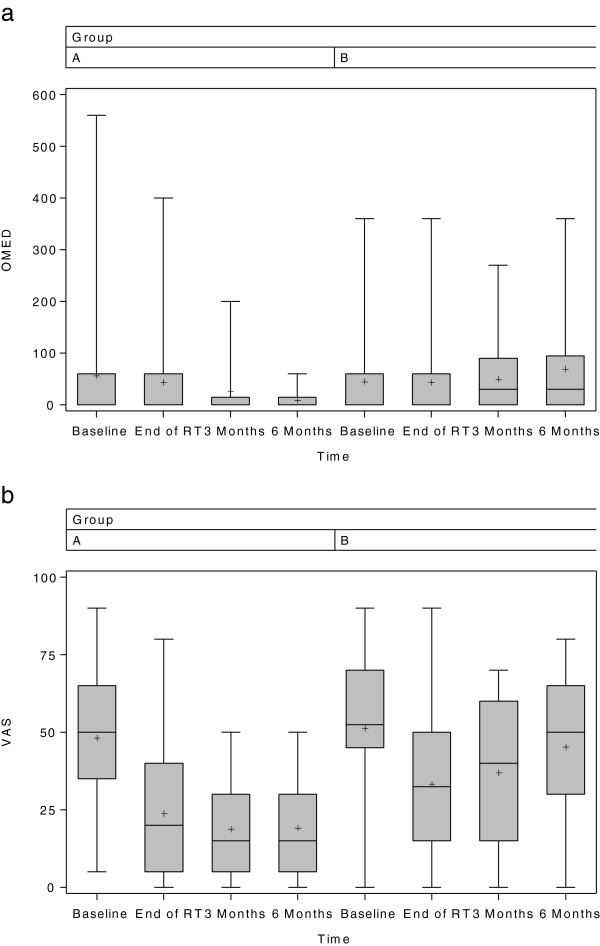
**OMED and VAS of both groups at measured points. a**. OMED of both groups at measured points (T0, T1, T2, T3). **b**. VAS of both groups at measured points (T0, T1, T2, T3).

In the course of time, the OMED decreased in arm A, but increased in arm B. After three months a positive trend in favor of arm A was discernible, and after six months the OMED was significantly lower (p = .018) (Figure 3a). At end of RT the mean VAS values showed a response to therapy in both groups, albeit significantly lower in arm A after three and six months (p < .001) (Table 3). In arm B the pain symptoms worsened up to six months after RT, while in the intervention group the VAS values remained virtually constant (Figure 3b). It was not possible to discern any difference between the groups in terms of neuropathic pain.

**Table 3:** Results of VAS, and concomitant medication during RT (day 1–10) and 12 weeks follow up

	**Intervention group (n = 30)**			**Control group (n = 30)**			
**Day 1–10 during RT**	**n**	**Mean**	**SD**	**n**	**Mean**	**SD**	**p-value**
VAS	30	192.5	126.9	30	394.4	223.0	<.001
CM	30	0.9	2.1	30	14.1	8.3	<.001
**Week 1–12 after RT**							
VAS	30	214.2	141.2	30	406.3	222.3	<.001
CM	30	2.7	4.5	30	17.7	10.3	<.001

*Abbreviations: VAS* visual analog scale, *CM* concomitant medication.

In Arm A, complete response and partial response rates at 12 weeks were documented in 4.6% and 63.6%, therefore 68.2% were responders (p = .172). After 6 months, 72.2% of patients were responders. Patients of control group showed a complete response and partial response at 12 weeks in 14.3% and 33.3%, thus 47.6% were responders. The rate after 6 months was only 22.2% (Table 4).

**Table 4:** Results of pain response

	**Intervention group**		**Control group**		
**After 12 weeks**	**n**	**%**	**n**	**%**	**p-value**
CR	1	4.6	3	14.3	0.196
PR	14	63.6	7	33.3	
PP	2	9.1	5	23.8	
SP	5	22.7	6	28.6	
Responders	15	68.2	10	47.6	0.172
Non-responders	7	31.8	11	52.4	
**After 6 months**					
CR	4	22.2	3	16.7	0.049
PR	12	66.8	3	16.7	
PP	1	5.5	4	22.2	
SP	1	5.5	8	44.4	
Responders	13	72.2	4	22.2	0.014
Non-responders	5	27.8	14	77.8	

*Abbreviations: CR* complete response, *PR* partial response, *PP* pain progression, *SP* stable pain.

## Discussion

The vertebral column is the main localization of bone metastases and pain is the most frequently reported symptom of an advanced stage of a tumor disease. Palliative RT is an effective means for the treatment of pain in patients with bone metastases of the spinal column [7,14] and will continue to remain the principal option for the treatment of painful bone metastases [15], with analgetics, systemic therapy, bisphosphonates, and minimal invasive surgery also playing their respective roles [7]. A non-pharmacological adjunctive therapy in the form of isometric training exercises of the autochthonous muscles has not yet been investigated and formed the basic concept of this novel pilot study.

Many other previously conducted studies have frequently used the pain response as the endpoint and presented this in the results. In most cases, a detailed report on the change in analgetics in the course of time was a weak point in the study design. The inclusion of the concomitant medication makes it possible to more exactly comprehend the therapeutic pain response to RT. Our results demonstrated a more substantial pain reduction during RT in the intervention group, whereas in arm B the pain symptoms increased again until six months after RT. Analgesic medication, measured by OMED, remained constant up to the end of RT in both groups; in arm A the requirement for analgetic medication decreased until up to six months after RT, while it significantly increased again in arm B (p = .018). Also the requirement for pain medication in arm A was significantly lower both during and after RT (p < .001).

Previous clinical studies have shown that tumor patients can indeed profit from physical training measures during and following medical treatment [4,16,17]. A Norwegian study of 355 patients were the RT-related pain response rates significantly lower after 2 months, but the OMED increases from 40 to 60 mg (p < .001) [18]. This increase in OMED could also be seen in our control group.

Patients affected by this condition are usually immobilized, primarily due to the risk of pathological fractures and the related danger of spinal cord compression. In our study we were able to demonstrate the positive effect of a training regimen to strengthen the muscles of the back as an adjunct to RT in reducing pain. Optimal treatment of skeletal metastases is complex, and a multidisciplinary approach is often needed. Analgetics should be administered in order to control symptoms additional to palliative RT. Some patients with bone metastases manifest bone pain with distinguishable neuropathic features [19]. It was not possible to demonstrate a difference between the two groups regarding neuropathic pain components. Single and multiple fractionated RT doses were equally effective in the palliative treatment of pain and the functional impact on extra- and intraspinal localizations [20], which is why the fractionating is not specified in the protocol. In the literature, the response to therapy has so far generally been expressed on the basis of pain [21]. In their study in 160 patients, Foro et al. showed a complete and partial response in the 30 Gy arm in 13% and 73% of the cases, respectively [13].

Chow et al. showed in their prospectively collected group of 518 patients the complete, partial, and overall response rates ranged from 21% to 25%, 26% to 30%, and 46% to 50% [22]. These results correlate with our control group after three months. In another work, Chow et al. showed in a systematic review of 25 randomized controlled trials complete response rates of 23% in the single fraction arm and 24% in the multiple fraction arm [23]. Our study presented complete response rates in the intervention group after three and six months of 4.6% and 22.2%, respectively, versus rates of 14.3% and 16.7%, respectively, in the control group; these were lower in both arms, although after six months a considerable 72.2% versus 22.2% of the patients were responders.

The effects of resistance training became apparent and significant in reduced pain after six months, which suggests a higher benefit for survivors.

Because all patients are in advanced stages of their cancer, 40% of the patients in either group were lost to follow-up due to progressive disease and subsequent death. Further limitations of the study are the relatively small sample size, the variety of primary tumors and patient conditions, and the exclusion of patients presenting with cervical spine metastases. For feasibility reasons, patients’ compliance with the training program in their homes was assessed only by relying on patient-completed documentation forms.

Among the strengths of the study are its randomized design and a relatively low drop-out rate, as well as standardized and specific measures to assess pain response among patients with bone metastases. This is, to our knowledge, the very first application of a resistance exercise program in patients with spinal metastases integrated in routine RT, to enhance their functional capacity and mobility, to reduce pain from spinal metastases.

## Conclusion

In this group of patients we were able to show that guided isometric resistance training of the autochthonous muscles can improve pain relief over a 6-months period in patients with stable spinal metastases. Importantly, the intervention was able to reduce OMED as well as concomitant pain medication. This exercise is a promising and effective therapeutic approach to reduce pain to patients suffering from spinal metastases. Large controlled trials are necessary to confirm these findings.

## Competing interests

The authors declare that they have no competing interests.

## Authors’ contributions

HR and JD developed and planned this trial. TB is responsible for statistical considerations/basis of the analysis. GO, MA, and TW estimated the stability of bone metastases. HR, MK, SR, MH, and IS performed the examinations and RT supervisions. HR and AG made the data collection. HR performed the physical exercise. All authors read and approved the final manuscript.

## Pre-publication history

The pre-publication history for this paper can be accessed here:

http://www.biomedcentral.com/1471-2407/14/485/prepub
